# Does it matter who writes down the feedback? A comparison of teacher- vs. student-completed clinical encounter cards during clinical rotations in veterinary studies

**DOI:** 10.3205/zma001170

**Published:** 2018-05-15

**Authors:** Heinz Hans Florian Buchner, Christoph Burger, Jan P. Ehlers

**Affiliations:** 1 University of Veterinary Medicine, University Equine Hospital, Vienna, Austria; 2 University of Vienna, Faculty of Psychology, Institute for Basic Psychological Research and Research Methods, Vienna, Austria; 3 University of Applied Sciences Upper Austria, Campus Linz, Department of Social Work, Linz, Austria; 4 Witten/Herdecke University, Faculty of Health, Chair of Didactics and Educational Research in Health Science, Witten, Germany

**Keywords:** Sustainable feedback, clinical rotations, clinical encounter card, feedback culture, personal responsibility, self-reflection, active feedback-seeking

## Abstract

**Objective: **Despite the fact that feedback (FB) provided by teachers to students is a recognised, effective teaching tool, successful use of feedback during clinical training depends on many factors. In addition to appropriate training and attitude of teachers, sustainable feedback requires an appropriate teaching culture and active commitment on the part of the students to receive, accept and use FB. This study examines the use of two different clinical encounter cards (CECs) during clinical rotation and investigates whether students take a more active part in the feedback process when using these cards. The objective of this study is to test whether it has a positive effect if students write down FB themselves and to verify this positive effect.

**Methodology:** 161 students in their 9th semester of veterinary studies each had to use two clinical encounter cards (types 1 and 2) during their rotations on 10 wards. For this, students had to ask teachers for FB before starting a clinical activity. The oral FB given by the teachers was either written down on the CEC by the teachers (CEC type 1) or by the students (CEC type 2). Furthermore, the students were asked to assess their own performance by means of anchor criteria and to evaluate the quality of the FB provided by the teachers. Based on the entries in the CECs submitted, the following indicators for both CEC types could be calculated: (1) FB quantity and quality (length and specificity), (2) differentiation of self-assessment, as well as (3) level of satisfaction with the FB provided by the teachers.

**Results: **With 2,377 CECs submitted, the mean CEC return rate was 74%. 99% of the cards showed positive FB, 69% contained constructive FB with suggestions for improvement, and 87% suggested specific next steps. On average, the FB written down by teachers was longer (12.4 versus 9.7 words) and more specific (1.9 versus 1.7 out of 3) than FB written down by students. Length and specificity decreased in the course of the semester. Neither the differentiation of self-assessment (proportion of differentiated entering of self-assessment) nor the students’ level of satisfaction with the FB differed between the two examined CEC variants.

**Conclusion: **The use of CECs across the cohort was successfully possible; however, the fact that students formulated and wrote down the FB themselves did not result in more comprehensive or more specific FB. Self-assessment and level of satisfaction with the teachers’ FB remained unchanged.

## Zusammenfassung

**Zielsetzung: **Feedback (FB) von Lehrenden an Studierende ist zwar ein anerkanntes, effektives Lehrmittel, ein erfolgreicher Einsatz in der klinischen Ausbildung aber von vielen Faktoren abhängig. Für ein nachhaltiges FB ist, neben einer entsprechenden Ausbildung und Einstellung der Lehrenden, eine entsprechende Lehrkultur nötig sowie ein aktives Engagement der Studierenden, um FB zu erhalten, anzunehmen und zu nutzen. In dieser Studie werden zwei verschiedene FB-Karten („clinical encounter cards“, CECs) während klinischer Rotationen eingesetzt und es wird untersucht, ob damit den Studierenden eine aktivere Rolle im Feedbackprozess gegeben werden kann. Als Hypothese wurde ein positiver Einfluss des eigenhändigen Niederschreibens von FB durch Studierenden postuliert und überprüft.

**Methodik: **161 Studierende im neunten Semester des Studiums Veterinärmedizin mussten während ihrer Rotationen auf 10 Stationen je zwei FB-Karten (Typ 1 und 2) nutzen. Dabei mussten die Studierenden vor einer klinischen Tätigkeit die zuständigen Lehrenden um FB bitten. Das von den Lehrenden mündlich gegebene FB wurde entweder von den Lehrenden (CEC Typ 1) oder von den Studierenden (CEC Typ 2) in die CEC aufgeschrieben. Weiters wurden die Studierenden aufgefordert, ihre eigene Leistung mittels Ankerkriterien zu bewerten und die Qualität des FB der Lehrenden zu beurteilen. Mittels der Eintragungen auf den abgegebenen CECs konnten die folgenden Indikatoren für beide CEC-Typen berechnet werden: (1) Quantität und Qualität des FB (Umfang und Spezifität), (2) Differenziertheit der Selbstbeurteilung, sowie (3) Zufriedenheit mit dem FB der Lehrenden.

**Ergebnisse:** Mit 2377 CECs lag die mittlere Rücklaufquote der CEC bei 74%. In 99% der Karten war positives FB eingetragen, bei 69% konstruktives FB mit Verbesserungsvorschlägen und bei 87% wurden konkrete nächste Schritte vorgeschlagen. Durchschnittlich schrieben Lehrende längeres (12,4 versus 9,7 Wörter) und spezifischeres FB (1,9 versus 1,7 von 3) auf als Studierende. Umfang und Spezifität nahmen während des Semesters ab. Weder Differenziertheit der Selbstbeurteilung (Anteil des differenzierten Eintragens der Selbstbeurteilung) noch die Zufriedenheit der Studierenden mit dem FB war zwischen den beiden untersuchten CEC-Varianten unterschiedlich.

**Schlussfolgerung:** Die jahrgangsweite Nutzung der CEC war erfolgreich möglich, allerdings ergab das eigenständige Formulieren und Eintragen des FB durch Studierende kein umfangreicheres oder spezifischeres FB, die Selbstbeurteilung und Zufriedenheit mit dem FB der Lehrenden war unverändert.

## 1. Introduction

### 1.1. The significance of sustainable feedback

Feedback (FB) provided by teachers to students, i.e. offering information on the performance of individual students when carrying out a specific activity with the aim to improve this performance in the future, has been described and recognised as didactic method for a long time [8], [12], [25]. Still, it is also well known for its implementation difficulties, lacking effectiveness and being perceived differently by teachers and students [3], [10], [19], [20], [23], [26]. These problems lead to a lower level of satisfaction with students and young physicists [6] or even to negative effects [14]. When analysing the various factors playing a role whether FB is successful or not, Hounsell [13] and Carless [6] introduced the term “sustainable feedback”, being based on dialogue processes and activities during which students are supported and informed about the actual task, but during which they shall also develop skills in self-regulating their performance during future tasks [6]. 

#### 1.2. The role of teachers, students and teaching culture in sustainable feedback

**Figure 1:**
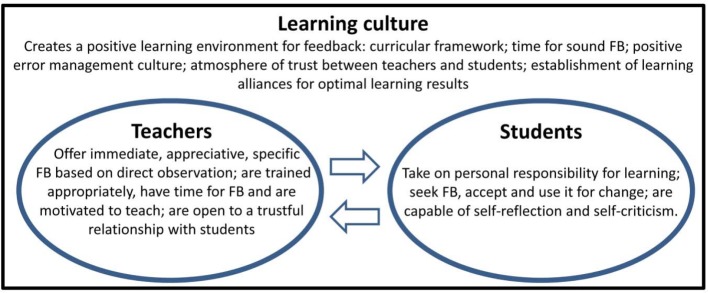
Sustainable feedback

The FB process and potential problem areas can be divided into three main parts (see Figure 1 (Fig. 1)):

1. Aspects of teachers being supposed to provide high-quality FB,
2. aspects of students being supposed to seek FB and use it accordingly, as well as
3. the relationship between both within a feedback team in a learning culture with FB as an integral part [6], [13], [27]].

Teachers are required to provide high-quality FB, which should be based on direct observation, provided as immediately as possible after observation, contain specific details and express an appreciative attitude [8], [26], [27]. This is only possible with appropriate training, enough time resources, sufficient teaching motivation and a trustful relationship with the students [1].

Students, as those receiving FB, also play an important part in a successful FB process. To ensure that FB is really helpful to students, they have to seek it (FB-seeking behaviour), accept and use it. Students weigh up pros and cons of FB. They are often worried that FB may do more harm than good to their ego and appearance [3]. Only if they realise this personal responsibility, as well as seek and initiate FB themselves, sustainable feedback will actually be possible [3], [7], [9], [16], [19]. In addition, the development of the ability to be self-critical and reflective while learning is another important step towards sustainable FB practice [21]. 

Such behaviour can be encouraged by transforming the FB process from “one-way FB” to “FB dialogue” [6]. This would be essential for a working FB culture with effective collaboration between teachers and students as a team. The curricular framework, time for sound FB, students’ confidence in the positive aims and helpful attitude of teachers, as well as the joint project of a learning alliance for optimal learning results are decisive factors for this learning culture [3], [6], [23], [27], [28].

#### 1.3. Background

The Equine Hospital of the University of Veterinary Medicine, Vienna, introduced a clinical encounter card (CEC), a proven method for stimulating FB culture [18], [22], during the summer semester 2014 in order to provide students with more FB. By means of these clinical encounter cards and before starting possible clinical activities, students asked the teacher for observation and feedback, which was then recorded on the CEC. During this pilot project, using the CECs indeed improved FB frequency and quality. However, known problems also occurred, such as the uncertain immediacy of FB provision, an ambiguous CEC design, as well as the students’ concern to put teachers, in particular older, experienced clinicians, under time-related stress by asking them for FB. The (purely formative) quantitative assessment of the students’ performance on a traditional grading scale on this CEC did not yield any valid values (only positive ratings) and has only been perceived as an impediment to FB [4]. These results provide the basis and starting point for this study.

#### 1.4. Study objective and hypotheses

The general objective of this study was the widespread introduction of an improved CEC into clinical rotations of veterinary studies, as well as the testing of options to render the students’ role in this FB process more active and encourage students to deal with feedback in terms of content. For one part, the initiation of FB by the students and, for the other part, the fact that the students write down the FB on the CEC themselves instead of the teachers may lead to dealing more intensely with FB and thus to more elaborate processing [24]. Moreover, it may also decrease the students’ concern about the time needed by teachers for providing FB, and increase the students’ awareness of their own accountability in the FB process. Such activation in the sense of “student-centred learning” would be an important step towards successful and more sustainable FB.

This study therefore aimed at testing the following hypotheses:

1. FB will get better with respect to quantity (length will increase) and quality (higher specificity; immediate provision of FB) if students write down FB on the CEC themselves;
2. The proportion of differentiated self-assessment of their own performance will become larger if students write down FB themselves; and
3. Students rate FB better and consider it being more helpful if they write down FB themselves.

## 2. Methodology

### 2.1. Participants

#### 2.1.1. Students

161 9^th^-semester students (82% female) participated in the project during clinical rotations of one week each on 10 wards of the animal hospital.

##### 2.1.2. Teachers

154 teachers (79% female) of the different clinics were available for teaching and giving FB. 24 of the teachers were completing their internship (first clinical postgraduate year), 96 juniors and 34 seniors (medical specialists; lecturers with postdoctoral qualification).

##### 2.1.3. Ethics committee

The study was examined by the ethics committees of the University of Veterinary Medicine and the University of Medicine, Vienna, and transferred to the Data Protection Commission. The latter approved the study in writing on 23 June 2015.

#### 2.2. Study design

**Figure 2:**
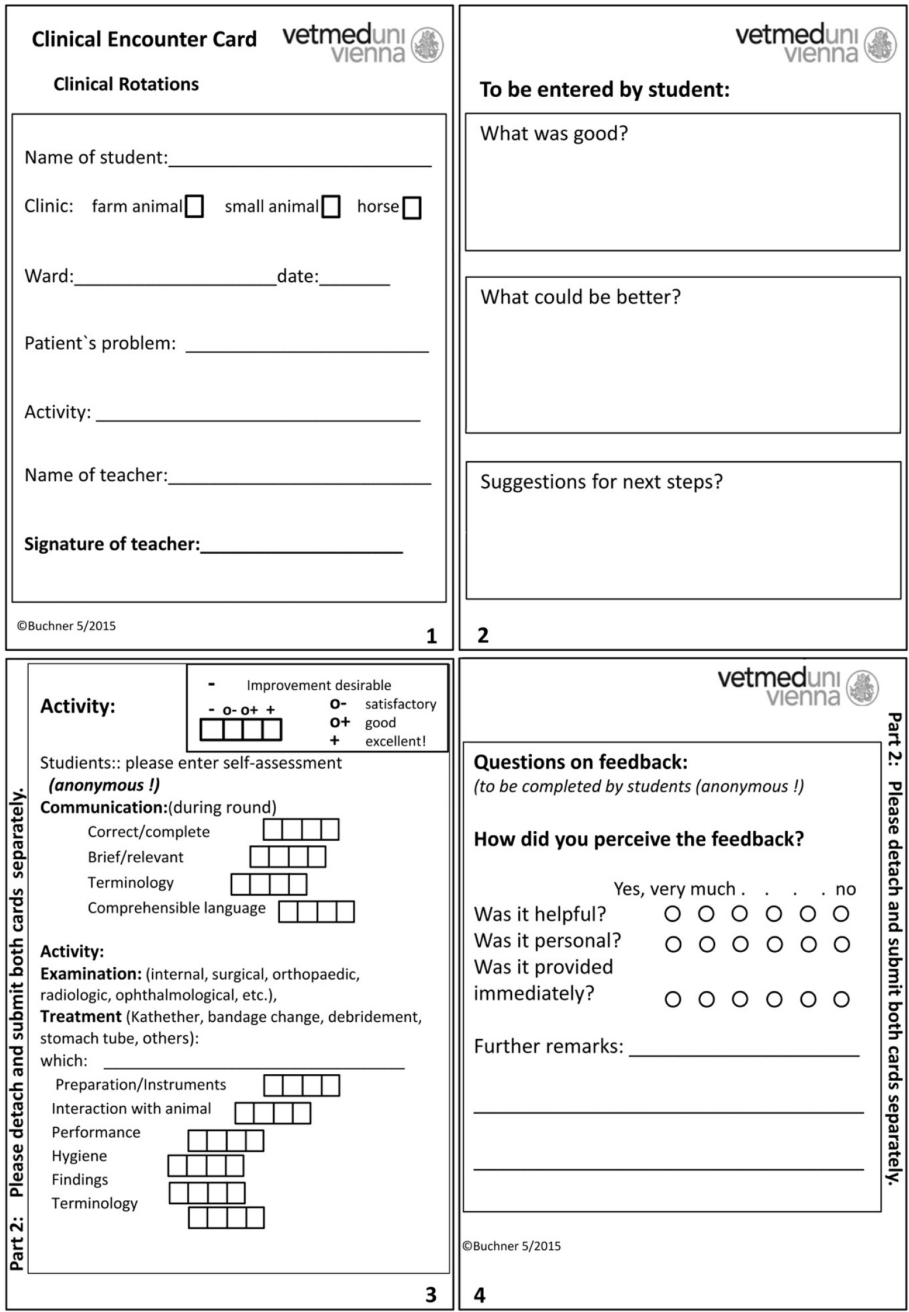
Type 2 clinical encounter card (FB entry by students)

Ten clinical wards of the animal hospital participated in the study. One of these wards (“W” clinic) offered the CECs to be completed voluntarily; all others required two completed CECs as part of the exercise. In order to prepare all teachers involved for giving FB to students, the teachers were offered 60-minute practical workshops. 52 teachers participated in the workshops. Teachers and students were provided with written information on the study and use of the CEC. At the beginning of each of the ten weeks, all students received two blank and different CECs: Type 1 and type 2 (see Figure 2 (Fig. 2)). Both types differed only on page 2 of part 1 (pages 1 and 2) by either asking the teachers (type 1) or the students (type 2) to write down the teacher’s oral FB. Page 1 contained general information on students, teachers and the activity performed. Part 2 of the CEC served to record the students’ self-assessment of their own performance after they had received the teacher’s feedback (page 3) and their impression of the usefulness of the FB (page 4), respectively. Part 1 and part 2 of the CECs (the latter being anonymous) were collected separately at the end of the practical week. Feedback had to be initiated by the students, the card to be handed over to the teacher and filled in after the activity had been performed and the FB provided. At the students’ request, more than two CECs could be handed out and used.

#### 2.3. Data analysis and statistics

Both parts of the CEC were submitted separately by the students in order to guarantee students the anonymity of their evaluation (unadulterated data quality through anonymity). The two parts could therefore not be assigned to each other and had to be evaluated separately.

The following data of part 1 (i.e. pages 1 and 2) was recorded and analysed in SPSS:

1. Descriptive data,
2. use of sections 1 to 3 (“What was good?”; “What could be better?”; “Suggestions for next steps?”),
3. the total number of words used, and
4. FB specificity.

The head of the study assessed the specificity on a four-digit scale (from 0 to 3), depending on whether no (0), 1, 2 or 3 and more different, specific content-related aspects had been addressed. In case of part 2 (i.e. pages 3 and 4), page 3 was assessed on a scale from 0 to 3, depending on the type of entry: 0=no entry; 1=all entries on the left side of the scale (“Improvement desirable”); 2=all entries on the right side of the scale (“Excellent”); 3=differentiated entries. 

The Likert scale values as well as available remarks on page 4 were collected and analysed. All parameters were evaluated descriptively, and the two groups “entry by teachers” or “entry by students” were compared statistically with respect to the individual parameters. Frequency differences were verified using the chi² test; depending on the quality, further comparisons were performed using t-test or Mann-Whitney u-test (non-parametrically: Likert values page 4). The correlation between the number of feedback words and their specificity was determined using Spearman’s rank correlation. The development over the different weeks regarding feedback length and specificity was determined using linear regression. The significance threshold was defined as α=0.05. The effect size according to Cohen (*d*) was calculated for the feedback quality (length, specificity) and the students’ remarks on the feedback (page 4). In addition, the impact of the teachers’ and students’ gender, as well as the teachers’ seniority on the quality parameters was examined. For this, a series of dual ANOVAs was performed using the number of words as dependent variable, as well as gender or seniority as first factor and the recording mode (teachers or students write down the FB) as second factor, respectively.

## 3. Results

### 3.1. Feasibility of CEC use

It was possible to introduce and use the CEC on all wards successfully. All in all, 2,377 CECs were evaluated, corresponding to 78% of the distributed cards of the obligatory CECs. In case of the “W” clinic, where the clinic management offered the CEC only as a voluntary tool, the rate of use and submission, respectively, was only 40.1% of the distributed cards.

#### 3.2. Quantity (increased length) and quality (higher specificity and immediacy)

The length of the entire feedback provided (number of words per CEC) was within a broad range of 0 to 84 words (see Table 1 (Tab. 1)). Likewise, the specificity of the documented feedback varied significantly between 0 (unspecific: e.g. “good”) and 3 or more aspects. Typical specific aspects of the FB on the performance of the activity were: technique, hygiene, procedure and speed, calmness during the performance or interaction with the horse. However, with a mean value of 11.1 words and a specificity of 1.8, the results of all clinics were very similar. Length and specificity correlate significantly (*r*=0.719, *p*<0.001). As expected, higher specificity generally correlates with a larger number of words. However, both quality parameters steadily decline over the course of the practical weeks (see Table 2 (Tab. 2)). In case of the teachers, the drop in values is somewhat more pronounced, coming from a higher starting level. They start out with approximately 14 words per CEC and end up with approximately 10 words. Similarly, teachers start with a specificity value of 2.1, declining to 1.5 in the end.

The feedback entered by teachers was significantly longer (12.4 versus 9.7) and more specific (1.9 versus 1.7) than the one written down by students (see Table 2 (Tab. 2)). This was primarily apparent from a large number of highly specific FB addressing three or more different aspects. This was also reflected by a positive effect size of Cohen’s *d*=0.37 for the number of words and *d*=0.21 for the specificity, respectively. 

The variance analysis yielded a significant effect only for the gender of teachers (*F*[1.2360]=10.4, *p*=.001, η*_p_*²=.004) and the combination of teachers and students (*F*[3.2356]=2.94, *p*=.007, η*_p_*²=.005), respectively: On average, female teachers gave more FB (*MV*=11.4 words, *SD*=8.0) than male teachers (*MV*=10.5 words, *SD*=6.1), in particular to female students (*MV*=11.5 words, *SD*=8.0).

#### 3.3. Differentiated self-assessment of one’s own performance 

Page 3 of the CEC for self-assessing one’s own performance was not completed at all by 2% of the students, at least uniformly completed by 52% with ticks only on the far left side of the scale (5.2% just “bad”) or only on the far right of the scale (46.6% just “excellent”) across all items. Only 46% of the students completed this page in a differentiated way, i.e. with at least one tick at another than an extreme position (far left or right). Entering the feedback personally (vs. teachers entering the feedback) had no impact on the degree of differentiated assessment (see Table 2 (Tab. 2)).

#### 3.4. Feedback assessment by students

In general, the feedback assessment on page 4 was predominantly positive. It was considered helpful (*MV*=1.8, *SD*=0.2) and very personal (*MV*=1.5, *SD*=0.2), and was also provided immediately in most cases (*MV*=1.6, *SD*=0.2). 15.6% of the students made further remarks, with 3.8% of them entering positive remarks in the CEC and 9.4% negative ones. 2.4% of the remarks concerned aspects other than the feedback, such as remarks on the conditions under which the activity was performed. Entering the feedback personally (vs. teachers entering the feedback) had no impact on the level of satisfaction with the feedback (see Table 2 (Tab. 2)).

## 4. Discussion

### 4.1. Feasibility and quality of the CEC intervention

The use of the CECs and handling of all organisational aspects of the study were possible on all wards without any problems. Despite the fact that 6 wards did not have any previous experience with structured FB, the more than 150 teachers and students showed much discipline in providing and writing down FB, as well as in submitting the CECs. The use of 78% of the required cards can be regarded as high; however, the missing 22% may be interpreted as insufficient consequence and conviction on the part of some teachers. The voluntary nature at the “W” clinic resulted in halving the number of cards submitted (40.1%), as well as in using the field “What could be better?” less: Only 47.1% made entries in this field compared to approximately 70% in general. This may indicate that mainly students being very self-confident and having less need for improvement in an activity asked for FB, in terms of self-selection. These students may therefore have used the CEC rather for strengthening their ego than for improving their skills [1].

The FB provided by individual teachers varied considerably with respect to length and specificity. Nevertheless, the assessment of the FB by the students resulted in very good ratings altogether. The FB was rated helpful with an average grade of 1.8 (out of 6 grades). The immediacy of the FB after the observed activity had been finished was also rated very positively at 1.6. Both grades are better than those achieved in the pilot project [4] and possibly reflect an improved training on the teachers’ part through repeated FB workshops. In any case, the CEC proves itself as a tool for regular and satisfactory FB of good quality for students.

Female teachers provided more comprehensive FB than male ones. This would be some kind of confirmation of the usual communication stereotypes, which are rather controversial though [15]. Nevertheless, this difference has a rather positive effect on the overall balance in light of this study’s gender distribution (teachers: 79f/21m; students: 81f/19m), ranging within the international average in the field of veterinary medicine nowadays. However, in the course of the semester the readiness to provide and write FB significantly decreased. The reason may be that the motivation for this instruction slowly wore off during this long, intensive practical training time, or may particularly relate to providing and writing down FB. It is also possible that the positive and motivating effect of FB to improve one’s performance by means of FB was not sufficiently noticeable. This could be traced back to the fact that the applicability of the FB was not practical enough, to a lack of repeatability, or to a missing learning alliance with an appropriate strategy for improving the performance [6], [23].

#### 4.2. Effects of teachers versus students writing down the FB

The evaluation of the question whether FB quantity and quality will be improved if students write down the FB themselves (versus teachers writing down the FB) clearly resulted in rejecting the hypothesis. Teachers definitely wrote down more comprehensive and more specific FB than students. In particular, highly specific FB with at least three commented aspects of the activity was recorded significantly more often by teachers (31.7%) than by students (19.7%). The teachers obviously took sufficient time for providing this FB to be in this lead. The question why students wrote down shorter and less specific FB cannot be answered reliably. One reason may be the additional effort for (and reluctance to) transferring the oral FB into writing on the CEC, even in case of FB aspects identified as being relevant. Some students described this effort as being superfluous in negative comments on the CEC. The personal writing down of FB by students requires the desired stimulation of the students, which was, however, apparently neither represented by longer FB texts nor by higher specificity. Alternatively, it could of course be imaginable in a positive sense that students deemed a few words being sufficient for recording the FB despite the fact that they regarded the FB as highly valuable. One option to analyse the students’ reasons for recording shorter FB is to set up several student focus groups and question them on their experience with the CEC and evaluation results.

The proportion of differentiated self-assessment by the students, interpreted by means of the degree of differentiated completion of the Likert self-assessment scale on page 3, was the same size in both trial variants (46% and 47%, respectively). The second hypothesis therefore had to be rejected as well. Of course, it is not possible to estimate reliably the actual reflection of the students on their own performance, apart from the entries on the CEC. The large proportion of sweepingly extreme values, i.e. ticking off everything on the left or the right side of the scale, can be seen as response bias by those just clicking through the answers and thus as resistance to the desired self-assessment and not as sincere reflection [11]. At any rate, this phenomenon was detectable to the same extent on both CEC variants in this study. 

The quality assessment of the FB by the students was remarkably similar in case of both CEC variants as well. Consequently, the third hypothesis that students will rate FB better if they enter FB themselves has to be rejected, too. 

#### 4.3. Sustainable feedback by means of the CEC?

A key concern of this study by comparing both CEC variants was to find an efficient method for stimulating students in terms of sustainable FB. Several aspects of this sustainable FB were initiated or tested by the CEC, such as FB quality, strengthening of student participation and self-reflection. The students’ role in particular is of substantial importance for untying the Gordian knot of effective FB [28]. The students’ personal responsibility and their willingness to actively enhance their learning progress are considered key aspects of sustainable feedback [6]. Seeking feedback (intrinsic) may be the first important step in this active learning process. However, the CEC was a prerequisite for completing the clinical exercises (extrinsic) and therefore not truly initiated by the students. On the one hand, the CEC thus seemed to be helpful because a lower threshold for seeking FB had to be overcome; on the other hand, as “compulsory exercise” it may also cause resistance and lead to a refusal of advice. At any rate, the obligation to use CECs has to be seen rather as an obstacle on the way to self-responsible learning, the objective of sustainable FB [6]. The proportion of 9.7% of negative comments shows this resistance. These comments were postulated frequently and in the same words by some clinical training groups. The proportion of negative comments was considerably lower at 4.7% at the “W” clinic, where students used the CEC on a voluntary basis. The more students develop an awareness of their right to receive feedback and the help feedback may provide, the sooner it can be expected that they will actively and readily seek FB. However, the proportion of differentiated self-assessment, if we wish to see it as a sign of positive stimulation, was not larger at the “W” clinic than the ones at the other clinics, despite the voluntary nature of seeking feedback. This problem of stimulating students to reflect on their performance is unfortunately known to be a great challenge [2].

The shorter text length and the lower specificity of the FB written down by the students themselves can indeed not be interpreted as a sign of successful student stimulation. The task of writing down and reflecting on FB, however, was new to the students and may be improved by building up competence in providing and seeking FB over several years. Such a development of competence can be expected with the introduction of the 2014 curriculum in Vienna, offering new communication seminars during which students learn and exercise how to provide and seek FB. This way, FB competence may be acquired gradually as a basis for active learning.

We were not able to use important elements of a sustainable FB culture in this study yet. Neither the two-stage assignment approach, i.e. intended repetition of activities after feedback has been received, nor the systematic use of introductory questions to the students were applied: “What do you think: What have you done well, what could you do better?” These methods would promote the FB dialogue and support self-reflection [6]. Hence, it would be possible to take further steps on the long way to a better FB culture in clinical training, where FB dialogue has its place and will become increasingly important in the tense atmosphere of all demands on students and clinicians.

## 5. Conclusions

By means of CECs and preparatory workshops for teachers, it is possible to achieve structured FB of sound quality for students. The students’ task to record the FB themselves neither leads to a more comprehensive and specific FB nor to a stimulation of differentiated self-assessment. Establishing a sustainable FB culture requires taking further steps in the medium term, such as curricular training in providing and receiving feedback, as well as a deliberate FB alliance between teachers and students, based on a dialogue of trust.

## Competing interests

The authors declare that they have no competing interests. 

## Figures and Tables

**Table 1:**
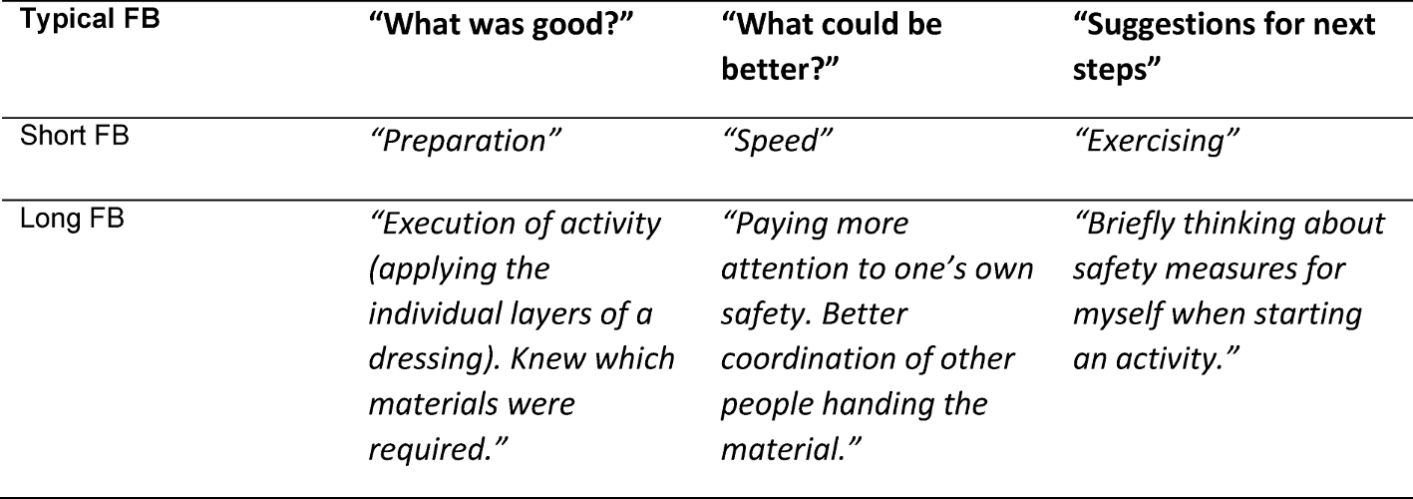
Typical examples of short and long feedback

**Table 2:**
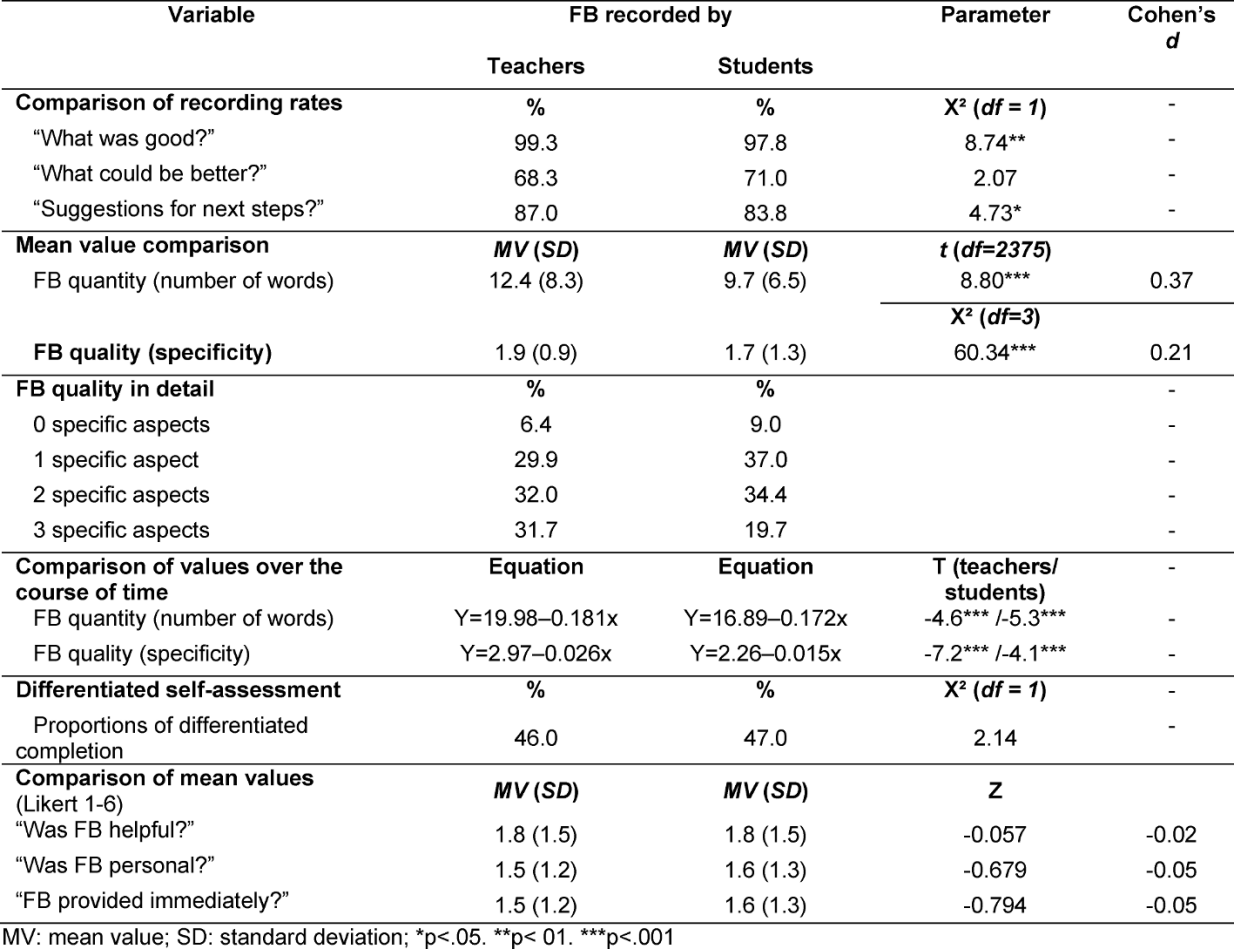
Quality of feedback recorded either by the teachers or by the students themselves and effects on the students’ self-assessment and their impression of the feedback quality
